# P29 Sinus tract formation after ultrasound guided biopsy

**DOI:** 10.1093/rap/rkae117.060

**Published:** 2024-11-05

**Authors:** Eman Elfar, Jennifer Hannah, Reuben Chua, Arti Mahto

**Affiliations:** King’s College Hospital, London, United Kingdom; Princess Royal University Hospital, London, United Kingdom; Flinders University, Adelaide, Australia; King’s College Hospital, London, United Kingdom

## Abstract

**Introduction:**

Synovial biopsy is a valuable diagnostic tool for investigating unexplained joint swelling, particularly when imaging and clinical evaluations are inconclusive in the context of suspected infection. This procedure is useful for diagnosing tuberculous (TB) arthritis. Whilst sinus tract formation is a known complication of TB, this is not always included in the consent process – particularly if the index of suspicion is low and previous screens have been negative. We highlight a case of sinus tract formation post-ultrasound guided synovial biopsy; ensuring informed consent and comprehensive evaluation with close monitoring post-procedure enhances patient outcomes and addresses complications early.

**Case description:**

A 71-year-old Caucasian male presented to his GP in November 2022 with a four-month history of progressive right knee pain, swelling, and reduced function. His medical history included anti-synthetase syndrome on mycophenolate, organizing pneumonia, pyoderma gangrenosum, IgG MGUS and hidradenitis suppurativa. He denied smoking, alcohol and IV drug use. Examination revealed a moderately swollen right knee, tenderness along the medial joint line without erythema or warmth. Initial knee X-rays showed early osteoarthritis (OA) signs and an effusion. An MRI in November 2022 revealed significant joint effusion, marked synovitis, and erosive changes in the proximal tibiofibular joint and medial tibial plateau. Inflammatory arthritis with possible secondary infection was suspected, he received intra-articular steroid injections with some relief.

By May 2023, after three ineffective intra-articular steroid doses, an ultrasound-guided synovial biopsy was performed, which was PCR-positive for TB despite a negative quantiferon. Consequently, anti-tubercular treatment (ATT) was initiated and immunosuppressants were ceased. Chest X-ray and CT were negative for pulmonary nodules but showed ground-glass opacities consistent with organising pneumonia. Further history revealed his low socioeconomic background and travel to Kenya, highlighting the risk for latent tuberculosis (TB) reactivation.

Post-biopsy, his symptoms worsened with increased joint pain, swelling, systemic symptoms, and a 4kg weight loss. In June 2023, he presented with increased swelling and reduced range of motion. Blood tests showed a normal white cell count but elevated CRP (105). An attempted knee aspiration found no fluid, but significant effusion and synovitis were noted. Later, he developed purulent discharge from a sinus on his knee, with cultures positive for acid-fast bacilli. Despite a surgical washout, severe knee pain and swelling persisted, and CRP peaked at 174. MRI showed severe joint damage, leading to a two-stage total knee replacement: initial resection arthroplasty with an antibiotic coated cement spacer, followed by definitive TKR after infection control.

**Discussion:**

Diagnosing and managing TB arthritis, especially in individuals with immunosuppressive conditions, is notably complex. The patient’s travel to TB-endemic regions likely contributed to latent TB, which reactivated due to immunosuppressive therapy. Despite a negative initial screening (IGRA), his history and clinical progression were consistent with TB reactivation.

This case underscores the critical need for rheumatologists to inform patients about the possibility of TB sinus formation post-biopsy, even in the absence of evident risk factors or a negative IGRA. Comprehensive evaluation and close monitoring are essential for effectively managing such patients.

The development of a sinus tract following synovial biopsy and the initiation of anti-tubercular therapy (ATT) was a significant complication in this case. This often indicates inadequate local disease control or secondary infection. The case highlights the importance of considering TB in the differential diagnosis of arthritis in patients with a history of immunosuppression, even when initial screenings are negative.

Given the patient’s worsening condition despite standard treatment, the decision for a two-stage knee replacement was prudent. This approach allows for thorough infection control before the definitive prosthetic implantation. The first stage involves resection arthroplasty and the insertion of a cement spacer, while the second stage, planned once the infection has cleared, involves the definitive total knee replacement. This method addresses both systemic and local manifestations of the disease, ensuring comprehensive patient care.

In conclusion, this case highlights the necessity of tailored management plans that consider the patient’s entire clinical picture, including their systemic and local disease manifestations. Rheumatologists and other healthcare providers must remain vigilant and proactive in their approach, ensuring that all potential complications are communicated to patients and managed appropriately. This strategy is vital for improving outcomes in patients with TB arthritis, particularly those with underlying immunosuppressive conditions.

**Key learning points:**

Key points

• **Consent for complications**: Rheumatologists performing ultrasound-guided or minimally invasive arthroscopic biopsies should inform patients about the risk of TB sinus tract formation, even if risk factors are absent and IGRA is negative.

• **Early and comprehensive evaluation**: Thorough history taking and consideration of latent TB reactivation in patients with prolonged immunosuppressive therapy are crucial.

• **Close monitoring**: Regular follow-up and monitoring of patients with TB arthritis are essential to detect and manage complications early.

• **Multidisciplinary approach**: Coordination between rheumatologists, infectious disease specialists, and orthopaedic surgeons is vital for optimal management of TB arthritis.

• **Tailored management plans**: Individualised treatment strategies, including surgical interventions, should be considered based on the severity of local and systemic disease manifestations.

